# Towards socially inclusive research: An evaluation of telephone questionnaire administration in a multilingual population

**DOI:** 10.1186/1471-2288-8-2

**Published:** 2008-01-31

**Authors:** Elizabeth Dormandy, Katrina Brown, Erin P Reid, Theresa M Marteau, SHIFT research team

**Affiliations:** 1 Department of Psychology (at Guy's), Health Psychology Section Institute of Psychiatry, King's College London, London, UK

## Abstract

**Background:**

Missing data may bias the results of clinical trials and other studies. This study describes the response rate, questionnaire responses and financial costs associated with offering participants from a multilingual population the option to complete questionnaires over the telephone.

**Methods:**

*Design: *Before and after study of two methods of questionnaire completion. *Participants and Setting: *Seven hundred and sixty five pregnant women from 25 general practices in two UK inner city Primary Care Trusts (PCTs) taking part in a cluster randomised controlled trial of offering antenatal sickle cell and thalassaemia screening in primary care. Two hundred and four participants did not speak English. Sixty one women were offered postal questionnaire completion only and 714 women were offered a choice of telephone or postal questionnaire completion.

*Outcome measures: *(i) Proportion of completed questionnaires, (ii) attitude and knowledge responses obtained from a questionnaire assessing informed choice.

**Results:**

The response rate from women offered postal completion was 26% compared with 67% for women offered a choice of telephone or postal completion (41% difference 95% CI Diff 30 to 52). For non-English speakers offered a choice of completion methods the response rate was 56% compared with 71% for English speakers (95% CI Diff 7 to 23). No difference was found for knowledge by completion method, but telephone completion was associated with more positive attitude classifications than postal completion (87 vs 96%, 95% CI diff 0.006 to 15). Compared with postal administration the additional costs associated with telephone administration were £3.90 per questionnaire for English speakers and £71.60 per questionnaire for non English speakers.

**Conclusion:**

Studies requiring data to be collected by questionnaire may obtain higher response rates from both English and non-English speakers when a choice of telephone or postal administration (and where necessary, an interpreter)is offered compared to offering postal administration only. This approach will, however, incur additional research costs and uncertainty remains about the equivalence of responses obtained from the two methods.

## Background

Missing data may bias the results of clinical trials and other studies, with low response rates compromising the validity of the findings [1,2]. Acceptable questionnaire response rates are considered to be in the range of 60–70%, with response rates of over 70% described as very good [3]. Self administered questionnaires sent and returned by post offer a cost effective way to obtain data from a large number of participants, but often has low response rates. A recent systematic review of methods to increase response rates to questionnaires identified a number of strategies such as shortening questionnaires, repeat mailing of questionnaires and telephone reminders [4]. Intensive reminders by telephone and post were the most effective, improving response rates by an average of 24%. Financial incentives have also been used to increase response rates [5]. For example a recent descriptive study reported that an unconditional payment of £5 increased the response rate from 78 to 88% [6].

Some studies comparing telephone and postal response rates from patients have found that response rates from questionnaires administered by telephone are higher [7] while others have found no difference [8]. Studies comparing responses from general practitioners have also shown varying results. One study reported a lower response rate for postal surveys than for telephone methods [9], while another found a higher response rate for postal surveys than for telephone methods [10]. Hocking and colleagues suggested that the lower telephone response rate in their study was because practice receptionists blocked telephone access to the general practioners who were the target of the survey. A consensus is emerging that a combination of direct contact and postal methods leads to higher response rates to questionnaires than the use of postal methods alone [8,11].

Two areas of uncertainty exist about the use of telephone compared with postal administration of questionnaires. First, research studies not only need a good response rate, but also representative responses from different socio-economic, demographic and clinical groups. It is not known whether postal and telephone methods of administering questionnaires affect responses differently from different socio-economic, demographic or clinical groups. Second, responses to questionnaire items administered by post or telephone need to be equivalent. Only two randomised studies have examined this. One found a difference in responses to the same question, while the other did not [8,12].

The observational study described here took place within SHIFT (Screening for Haemoglobinopathies in the First. Trimester), a cluster randomised controlled trial assessing the feasibility, effectiveness and acceptability of offering antenatal Sickle Cell and Thalassaemia (SCT) screening in primary care (ISRCTN00677850) [13]. The trial was set in two inner city UK Primary Care Trusts (PCTs) with a large number of people from minority ethnic groups and with high levels of material and social deprivation. The questionnaires had been designed to assess informed choice about antenatal SCT screening in a population with low levels of literacy [14].

At the start of the SHIFT trial, participants were posted questionnaires (in one of twelve languages, selected according to information provided at trial consent) to complete at home and return by post. Initial monitoring indicated that this method gave an unacceptably low response rate. Based on a literature search we developed a second strategy that was more likely to achieve a high response rate in a multilingual population namely, offering participants the opportunity to complete a questionnaire over the telephone with a researcher and an interpreter, if necessary. Participants offered the second strategy were also offered postal completion. This paper compares (i) the response rates of offering participants a choice of telephone and postal completion methods with those of offering postal completion only; (ii) the responses obtained from the method selected ie postal or telephone completion; (iii) the financial costs to the research team of offering postal and telephone administration of questionnaires in order that reliable estimates can be included in future research cost estimates.

## Methods

### Study design

The study has a "before" and "after" design, comparing two methods of questionnaire completion. Eligible participants were asked by their GPs if the research team could contact them to invite them to take part in the trial evaluation and, if so, in which language they would like to be contacted. Women who agreed to be contacted by the research team were contacted in a language identified by the GP as the woman's preferred language, using a telephone interpreter if appropriate, and informed about the trial. For women consenting to take part two methods of questionnaire completion were used:

#### Postal completion only

A written questionnaire was sent in one of twelve languages and women were asked to return it using a freepost envelope. Up to two reminders to return the completed questionnaire were sent.

#### Choice of telephone or postal completion

During a telephone conversation to recruit interested women to the trial, women were offered a choice of telephone or postal completion of the questionnaire: (a) telephone completion – the questionnaire was read to women over the telephone in their preferred language using a telephone interpreter if necessary. Women were offered the choice of completing the questionnaire at the time consent was sought or at a later stage. A script was developed for telephone administration of the questionnaire detailing exactly how to present response options. Women who started, but did not finish the questionnaire over the telephone, were sent a written questionnaire in the post. Up to two reminders to return the written questionnaire were sent.

(b) postal completion – as described above.

### Setting

Twenty five general practices in two UK Primary Care Trusts (PCTs). The study represents the first eleven months of the evaluation phase of a cluster randomised controlled trial of offering antenatal SCT screening in primary care [13]. The method of recruiting and retaining representative practices to the trial is described elsewhere [15]. A universal screening policy was operating during the data collection period, that is, antenatal SCT screening was offered to all pregnant women regardless of their ethnicity or family origin [16]. It is estimated that about 6% of pregnant women in the two PCTs carry a significant haemoglobin variant [16]. The two PCTs are ranked among the most deprived in England (sixth and 13th out of 354 boroughs) and have about 40% of their total populations from minority ethnic groups [17]. The questionnaire comprised 32 items, including four items assessing attitudes towards antenatal SCT screening and ten items assessing knowledge about antenatal SCT screening [14]. It is estimated the questionnaire took participants between five and ten minutes to complete on their own.

### Participants

Seven-hundred and sixty-five pregnant women consenting to take part in a questionnaire evaluation of antenatal sickle cell and thalassaemia screening. The only measure of social group available was "English speaking" or "non English speaking". Women were classified by their GP into these groups, based on whether the woman required an interpreter to speak to a member of the research team. In such cases the GP indicated the woman's preferred language. There were 571 women in the English speaking group and 204 women in the non English speaking group. Sixty one women were asked to complete and return questionnaires using postal completion only. Seven hundred and fourteen women were offered a choice of completing the questionnaire using telephone or postal completion. The uneven group sizes occurred because the response rate from sending questionnaires by post was recognised as unacceptable early in the evaluation phase of the trial and so was discontinued.

### Measures

We report on

(i) response rates for women offered postal completion only compared with women offered a choice of completion methods

(ii) questionnaire responses on two sub scales of the questionnaire

• attitudes towards undergoing antenatal SCT screening based on four items. Positive and negative attitudes were defined by the midpoint of the scale, with scores above 12 denoting positive attitudes towards undergoing the test [18].

• knowledge about undergoing antenatal SCT screening based on ten items. Good and poor knowledge were defined by the midpoint of the scale, with scores above 5 denoting good levels of knowledge. [14]

(iii) costs associated with administering postal and telephone questionnaires were estimated by measuring the time taken to (a) administer 50 questionnaires over the telephone and (b) prepare and post 50 questionnaires. Costs were based on the means of these two sets of data. Interpreter costs were estimated from (a) charges made by a commercial interpreting company (£1.50 per minute plus 17.5% tax) and (b) the mean length of time taken to complete 50 interpreted questionnaires over the telephone.

Ethical approval was granted for the study (05/Q0501/36).

## Results

The response rate for women offered postal completion only was 26% compared with 67% for women offered a choice of telephone or postal completion (41% difference, 95% Confidence interval of difference (CI Diff) 30 to 52) (Table 1) [19]. Women who spoke English had a higher response rate to a choice of completion methods compared with women who did not speak English (71% vs. 56%, 95% CI Diff 7 to 23). There were insufficient women who did not speak English and were only sent questionnaires by post to make a valid comparison with women who spoke English and were only sent questionnaires by post i.e. between English and non English speakers who were not offered a choice of completion methods.

**Table 1:** Response rate by questionnaire completion method

All women consenting to take part in the trial evaluation (n= 775)	Postal completion only n = 61	Choice of telephone or postal completion n= 714
*Women who do and do not speak English*		
Questionnaires Administered	61	714
Questionnaires Received	16	476
**Response rate, (%, 95% CI)**	**26% (16 to 39)**	**67% (63 to 70)**

*Women who speak English (n = 571)*		
Questionnaires Administered	55	516
Questionnaires Received	15	365
**Response rate, (%, 95% CI)**	**27% (16 to 41)**	**71% (67 to 75)**

*Women who do not speak English (n= 204)*		
Questionnaires Administered	6	198
Questionnaires Received	1	111
**Response rate, (%, 95% CI)**	**17% (0.4 to 64)**	**56% (49 to 63)**

The preferences and response rates of the sub-group of women offered a choice of completion methods are shown in Table 2. Among the women offered a choice of completion methods, 58% chose telephone completion (416/714 95% Confidence interval (CI) 55 to 62). The response rate for women choosing telephone completion was 98% compared with 23% for women choosing postal completion (75% difference, 95% CI Diff 70 to 80). Eight women, having opted for telephone completion, did not complete the questionnaire. Questionnaires were sent in the post to these women. Of these eight, none was completed or returned. Women who did and did not speak English had similarly low response rates to postal questionnaires (23% vs. 23%, 95% CI Diff -10 to +10%). While both groups had very high response rates to telephone completion, women who spoke English had slightly higher rates (99% vs. 94%, 95% CI Diff 0.7 to 11%).

**Table 2:** Preferences and response rates for women offered a choice of questionnaire completion method.

All women offered a choice of completion method (n = 714)	Women choosing postal response	Women choosing telephone response
*Women who do and do not speak English*		
Questionnaires Administered	298	416
Questionnaires Received	68	408
**Response rate, (%, 95% CI)**	**23% (18 to 27)**	**98% (96 to 99)**

*Women who speak English (n = 516)*		
Questionnaires Administered	193	323
Questionnaires Received	44	321
**Response rate, (%, 95% CI)**	**23% (17 to 29)**	**99% (98 to 100)**

*Women who do not speak English (n= 198)*	*Women who do not speak English*	*Women who do not speak English*
Questionnaires Administered	105	93
Questionnaires Received	24	87
**Response rate, (%, 95% CI)**	**23% (15 to 32)**	**94% (87 to 98)**

**Figure 1:**
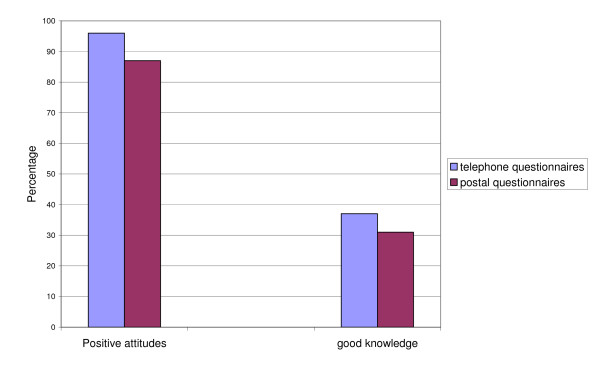
The percentage of women identified with positive attitudes and good knowledge by questionnaire completion method.

More women were identified as having positive attitudes towards undergoing antenatal SCT screening using telephone questionnaires than using postal questionnaires (96% vs 87%, 95% CI Difference 0.006 to 15%). There were no differences  in the proportion of women identified with good knowledge about the test  using telephone or postal questionnaires ( 37% vs 31% 95% CI diff -6 to 17%  ) (Figure 1)

Telephone questionnaires were completed in twenty languages other than English. Postal questionnaires were completed in nine languages other than English.

### Costs

The costs associated with the administration of questionnaires are:

(i) The cost of researcher time to administer telephone questionnaires. The mean length of time to administer the the 32-item questionnaire questionnaire over the telephone was fifteen minutes. This equates to £4.50 of researcher time.

(ii) The cost of interpreters and researcher time to administer telephone questionnaires for non-English speakers. The mean length of time to administer a questionnaire via an interpreter over the telephone was thirty five minutes. This equates to approximately £10.60 of researcher time and £61.60 for the interpreting services. Thus, in total it cost £72.20 to administer a 32-item questionnaire over the telephone using an interpreter.

(iii) The cost of researcher time to prepare and post questionnaires. The mean length of time to prepare and post a questionnaire was two minutes. This equates to approximately 60 pence of researcher time.

Compared with administering questionnaires by post, administering a telephone questionnaire cost an additional £3.90 for English speakers and £71.60 for non-English speakers.

## Discussion

The results of this study suggest that offering a choice of telephone or postal completion methods can result in a dramatic increase in response rates compared with postal completion alone. This study was not a  randomised trial and there could be other explanations for the observed  effect. The intervention was complex. It included offering a choice, and offering a choice of two methods that differed in several ways. While we cannot exclude the possibility that the mere offer of a choice was responsible for the effect, the pattern of results suggests that it is the opportunity to complete questionnaires by telephone that is crucial. Telephone completion compared with postal completion allows for ready translation to more languages and provides social support to complete the questionnaire. Perhaps most importantly it removes the reading obstacle to questionnaire completion thereby allowing the estimated 20–25% of the UK population who are functionally illiterate [20] to participate in the research process.

The increased response rate obtained by administering questionnaires by telephone raises two questions: first, did the increase in response rate vary by social group; and second, were responses obtained by telephone different to those obtained by post. Regarding differential response rates across social groups, the response from those offered a choice in this study was higher for English speakers than for non English speakers. The lack of data from non responders means, however, that we are not able to assess how representative the responders are of the telephone or postal groups overall.

The data do not identify a reason why non-English speakers were less likely to opt for completing the questionnaire by telephone than were English speakers. It may reflect a failure of the trial to engage non-English speakers in the research process. Alternatively, it may reflect cultural differences in, for example, willingness to talk to strangers over the telephone. Further work is required to understand why non-English speaking women were less likely to choose to complete questionnaires by telephone than were English speaking women. Understanding this may allow telephone administration to be offered in ways that increase acceptability, and hence, use for non-English speakers and thereby increase response rates further.

Comparing responses from the two modalities revealed differences in assessed attitudes: women completing the questionnaire over the telephone were more likely to be classified as having positive attitudes towards undergoing antenatal SCT screening than women completing the postal questionnaire. There were no differences in knowledge by completion modality. One explanation is that the difference may be due to the way the questions were asked or social desirability. Alternatively it may be because the study was not randomised. That is women who opted to complete the questionnaire over the telephone had more positive attitudes towards undergoing the test than women who completed the questionnaire by post.

There are financial costs involved in offering women a choice of telephone or postal questionnaire completion methods. These include the researcher time to administer the questionnaire over the telephone as well as the cost of using telephone interpreters. The use of telephone interpreters did not negate the need (or cost) for translating written materials. Research budgets should include funding to cover all these costs.

There are potential problems associated with administering questionnaires over the telephone. The person administering the questionnaire needs to be trained to ensure that the potential participant does not feel under any pressure to participate in the evaluation or to complete the questionnaire. Training is also required to ensure that questions are not asked in a leading way i.e. in a way likely to guide respondents to answer in a particular way.

For women who opted to complete the questionnaire by telephone, the use of interpreters did not appear to pose any problems. The use of telephone interpreters allows greater flexibility than using written translations because the languages required do not require specifying in advance. However the use of telephone interpreters does not allow for the use of quality control procedures such as back translation that are available with written translations.

There has been some debate about the use of translation services within the NHS which cost in the region of £55 million per annum [21]. It has been argued that the use of such services may compound rather than ameliorate the health problems of non-English speakers by reducing the need for them to learn English and thereby implicitly encouraging non-English speakers to remain outside of the dominant culture [22]. Others have argued that the lack of translation services results in poorer healthcare for non-English speakers [23]. Whilst this debate is likely to continue within the context of service provision, it is important to acknowledge that this debate is not applicable in research settings. A prerequisite for reliable trials is that trial outcomes are obtained for all participants and are representative of the population in general [2]. They therefore need to include people who do as well as those who do not speak English.

### Strengths and limitations

The strength of this study is that it illustrates the acceptability and feasibility of offering respondants the option of completing questionnaires over the telephone with and without interpreters. A weakness of the study is that the effect of offering telephone administration, although large, is based on observational data, and so uncertainty remains about a causal link between offering women an opportunity to complete questionnaires over the telephone and the observed increase in questionnaire response rates. Whilst the study took place in areas with high levels of social deprivation, individual level markers of social deprivation were not available. It is therefore unknown by how much, if at all, offering telephone administration of the questionnaire increased the percentage of participants with high levels of material and social deprivation. The results also raise questions about the equivalence of responses to questions obtained using the two methods. More research is needed to determine if this is due to differences in types of people responding or differences in demand characteristics of the two methods.

## Conclusion

Studies requiring data to be collected by questionnaire may obtain higher response rates from both English and non-English speakers when a choice of telephone or postal administration and where necessary, an interpreter, is offered compared to offering postal administration only. This approach will, however, incur additional research costs and uncertainty remains about the equivalence of responses obtained in the two methods.

## Competing interests

The author(s) declare that they have no competing interests.

## Authors' contributions

ED participated in the design of the study and performed the statistical analysis and drafted the manuscript; KB participated in the design of the study and conducted the telephone interviews; ER participated in the design of the study and helped with drafting the manuscript; TMM conceived of the study, participated in it's design and co-ordination and helped to draft the manuscript. All authors read and approved the final manuscript

## Pre-publication history

The pre-publication history for this paper can be accessed here:


